# Weight Gain Associated with COVID-19 Lockdown in Children and Adolescents: A Systematic Review and Meta-Analysis

**DOI:** 10.3390/nu13103668

**Published:** 2021-10-19

**Authors:** Tu-Hsuan Chang, Yu-Chin Chen, Wei-Yu Chen, Chun-Yu Chen, Wei-Yun Hsu, Yun Chou, Yi-Hsin Chang

**Affiliations:** 1 Department of Pediatrics, Chi Mei Medical Center, Tainan 71004, Taiwan; cych07324@gmail.com (W.-Y.C.); drchen9090@gmail.com (C.-Y.C.); wyhsu17@gmail.com (W.-Y.H.); a2728809@gmail.com (Y.C.); 2 Department of Pediatrics, Chi Mei Medical Center, Chiali, Tainan 72263, Taiwan; ruby721222@hotmail.com (Y.-C.C.); quashow@gmail.com (Y.-H.C.)

**Keywords:** COVID-19, lockdown, body weight, BMI, children, adolescents

## Abstract

Background: Lockdown is an effective nonpharmaceutical intervention to reduce coronavirus disease 2019 (COVID-19) transmission, but it restricts daily activity. We aimed to investigate the impact of lockdown on pediatric body weight and body mass index (BMI). Methods: The systematic review and meta-analysis were conducted following the Preferred Reporting Items for Systematic Reviews and Meta-analysis (PRISMA) statement. Four online databases (EMBASE, Medline, the Cochrane Library and CINAHL) were searched. Results: The pooled results showed that lockdown was associated with significant body weight gain (MD 2.67, 95% CI 2.12–3.23; *p* < 0.00001). The BMI of children with comorbidities or obesity did not change significantly. The BMI of general population was significantly higher during lockdown than before the pandemic (MD 0.94, 95% CI 0.32–1.56; *p* = 0.003). However, heterogeneity was high (I^2^ = 84%). Among changes in weight classification, increases in the rates of obesity (OR 1.23, 95% CI 1.10–1.37; *p* = 0.0002) and overweight (OR 1.17, 95% CI 1.06–1.29; *p* = 0.001) were reported. Conclusions: Our meta-analysis showed significant increases in body weight and BMI during lockdown among school-age children and adolescents. The prevalence of obesity and overweight also increased. The COVID-19 pandemic worsened the burden of childhood obesity.

## 1. Introduction

Coronavirus disease 2019 (COVID-19) has changed both global health and economic activities. Movement restrictions and lockdowns may temporally slow the spread of the virus. However, prolonged stringent restrictions increase unemployment and mental stress, which may outweigh the benefits of community-based mitigation.

In addition to its health and economic effects, COVID-19 has a great impact on people’s lifestyles, including activities such as remote education, eating behaviors and physical activity [1,2,3]. These changes have direct effects on body weight. In a recently published meta-analysis, significantly increased body weight and BMI were observed during the post-lockdown period in the adult population [4]. However, this result was inconclusive when subgroup analysis was performed according to different age groups. Another study also reported body weight loss in the elderly, indicating the potential issue of malnutrition and sarcopenia [5]. The pros and cons of lockdown among different populations are uncertain.

Children are a unique population affected by the COVID-19 pandemic. The probability of children developing severe disease is low [6]. Current evidence shows that the transmission rate is low in schools and childcare facilities [7,8,9]. Nevertheless, unlike businesses, educational facilities usually close during the first wave of an outbreak. School closures disrupt learning and change the lifestyles of children. Increased stress and anxiety during lockdown or quarantine have also been indicated [10]. Interactions of psychosocial factors might further contribute to weight gain and obesity in childhood. However, current data are limited. Therefore, we aimed to investigate the impact of lockdown on body weight in the pediatric population.

## 2. Materials and Methods

### 2.1. Search Strategy

**Figure 1:**
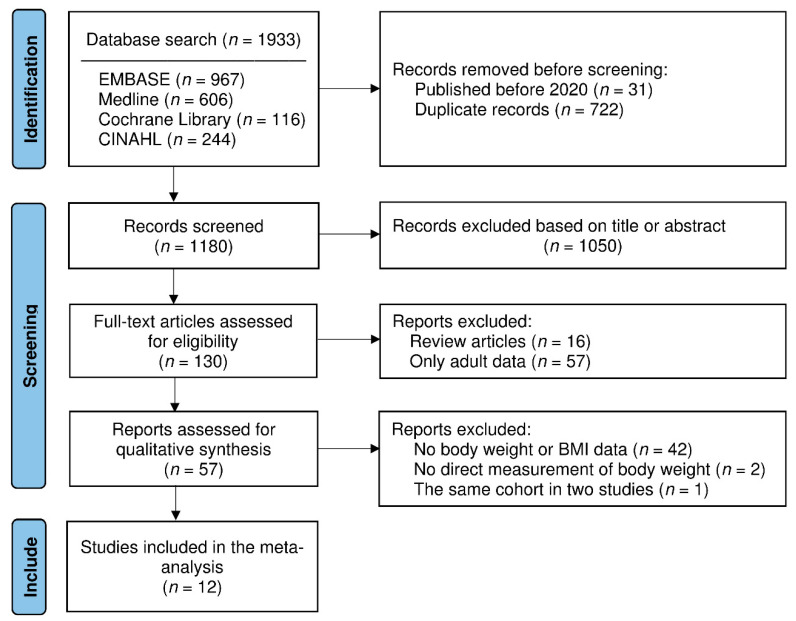
Flow diagram of article identification for inclusion in the qualitative and quantitative analysis.

The present study was conducted following the recommendations of the Preferred Reporting Items for Systematic Reviews and Meta-analysis (PRISMA) statement. We performed a systematic literature search in the following four major electronic databases: EMBASE, Medline, the Cochrane Library and CINAHL. We used the following keywords for the search: “COVID-19”, “lockdown”, “body weight or body mass index”, “diet” and “lifestyle”. We provide a detailed search strategy in Supplementary S1 and Figure 1. No language restriction was applied. Google Scholar was searched manually for possible missed articles. The last search was conducted on 9 October 2021.

Three reviewers (THC, YCC and WYC) independently screened all the titles and abstracts for eligibility. Studies were eligible for inclusion if they met the following criteria: (1) data on body weight or BMI before and during/after the lockdown; and (2) children or adolescents included in the study population. The exclusion criteria were as follows: (1) publication before 2020 because the formal report of severe acute respiratory syndrome coronavirus 2 (SARS-CoV-2) had not been released; (2) preschool-age children included in the study population because some of them had not attended school and were thus less affected by lockdown; (3) only descriptions of body weight, such as “weight gain” or “weight change”, without anthropometric measurements; or (4) the enrolled population was diagnosed with COVID-19 during the study period.

### 2.2. Data Extraction

After full-text screening for eligibility and review, two reviewers (THC and YCC) independently extracted data. Disagreements were resolved by discussion or consultation with a third reviewer (WYH). The following data were extracted from each study if available: author information, journal, year of publication, country, study period, lockdown duration, study population, age, sex, health status, weight status (normal weight, overweight or obese), method of body weight measurement, body weight and BMI. If the same cohort was reported in more than one publication, the study with the largest sample that met the inclusion criteria was included.

### 2.3. Quality and Risk of Bias Assessments

The quality of nonrandomized studies included in the meta-analysis was assessed using the Newcastle–Ottawa Scale (NOS) by two reviewers (YHC and YC). The maximum score for each study was nine, and articles with poor quality (scores = 0–3) were excluded. The detailed scoring table is shown in Supplementary S2.

### 2.4. Outcomes and Data Analysis

The results from the included studies were pooled for further analysis. Due to inherent differences in the study populations, we also performed subgroup analysis if heterogeneity or heterogeneous groups existed. The outcomes measured in our studies included body weight or BMI change during lockdown. In addition, we compared the proportion of overweight or obese individuals in the studied population before and during the lockdown period.

We calculated odds ratios (ORs) for dichotomous outcomes (obesity or overweight) and mean differences (MDs) for continuous data (body weight, BMI), with their 95% confidence intervals (CIs), using random-effects models in this meta-analysis. We used Review Manager software version 5.4 (Nordic Cochrane Centre, Cochrane Collaboration) for the analysis, and we conducted a meta-analysis when at least two studies with available data reported the same outcome. The I^2^ statistic was calculated to measure heterogeneity across the included studies. I^2^ > 50% and *p* < 0.10 were considered to indicate substantial heterogeneity. Publication bias was not assessed for any outcome because fewer than 10 studies were available.

## 3. Results

### 3.1. Study Selection

Figure 1 shows the flow diagram of the study selection process. In total, 1933 (967 from EMBASE, 606 from Medline, 116 from the Cochrane Library and 244 from CINAHL) records were identified through database searching. After removing 722 duplicates and 31 articles published before 2020, the titles and abstracts of 1180 articles were screened, and obviously irrelevant articles (*n* = 1050) were excluded. The remaining 130 articles were retrieved for full-text assessment. We excluded 16 review articles and 57 articles describing only adult populations. Fifty-seven articles were then selected for qualitative synthesis, and the following articles were excluded: 42 articles without body weight or BMI reports; 2 articles without measurement of body weight; and 1 article that described the same cohort that was presented in another study. Finally, we extracted data from 12 articles for the meta-analysis.

### 3.2. Study Characteristics

Table 1 summarizes the characteristics of the current literature associated with body weight or BMI change during lockdown in children. The 12 included studies were conducted in 8 different countries, and the lockdown duration ranged from 1–5 months. Among the 12 studies, 3 included children with type 1 diabetes mellitus, 2 included children with obesity or overweight, and 7 included relatively healthy children. Methods of body weight measurement were either direct measurement or online questionnaires with self-reported body weight.

### 3.3. Changes in Body Weight during Lockdown

**Figure 2:**
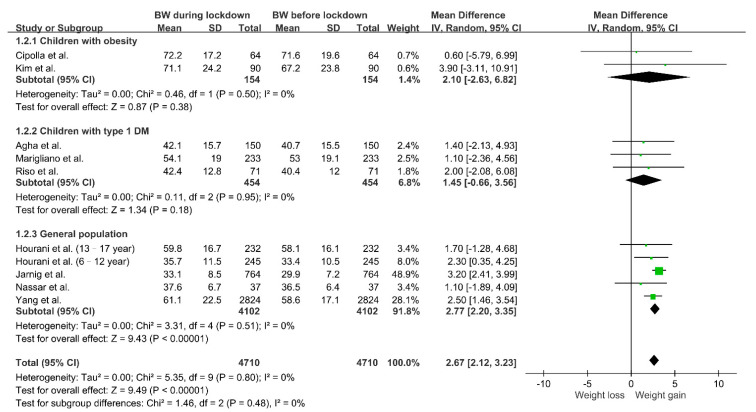
Changes in body weight during lockdown in children. Study populations were further divided into three groups (children with obesity, children with type 1 diabetes mellitus and children without comorbidities), and subgroup analysis was performed. Abbreviations: BW, body weight; DM, diabetes mellitus.

As shown in Figure 2, we explored the relationship between COVID-19 lockdown and body weight change. The pooled result showed that social restriction was associated with body weight gain (MD 2.67, 95% CI 2.12–3.23; *p* < 0.00001) with no heterogeneity (I^2^ = 0%, *p* = 0.80). However, because of potential population differences, we performed subgroup analysis according to health status. In children with type 1 diabetes mellitus or obesity, there was a trend toward body weight gain during lockdown without statistical significance. In the relatively healthy population, we observed substantial body weight gain during the time that the restrictive measures were in place (MD 2.77, 95% CI 2.20–3.35; *p* < 0.00001).

### 3.4. Changes in BMI during Lockdown

**Figure 3:**
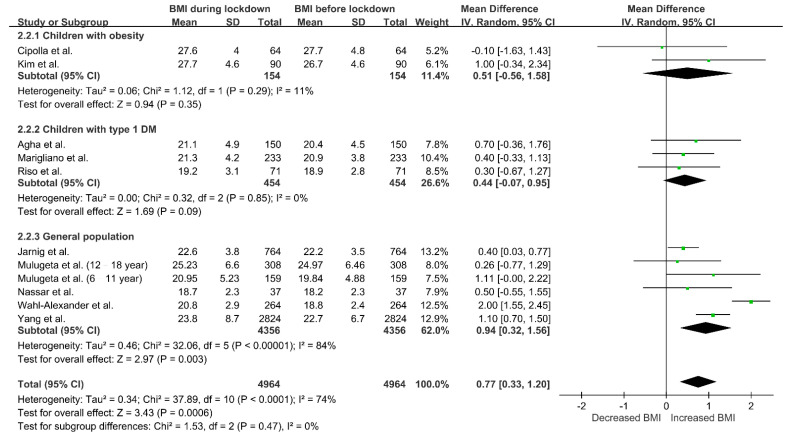
Changes in BMI during lockdown in children. Study populations were further divided into three groups (children with obesity, children with type 1 diabetes mellitus and children without comorbidities), and subgroup analysis was performed. Abbreviations: BMI, body mass index; DM, diabetes mellitus.

Figure 3 shows the relationship between BMI and lockdown. In general, the BMI of children increased during lockdown (MD 0.77, 95% CI 0.33–1.20; *p* = 0.0006), but the heterogeneity among studies was high (I^2^ = 74%, *p* < 0.0001). We performed subgroup analysis according to study population. In children with type 1 diabetes mellitus or obesity, BMI did not significantly change during the period with strict restrictions. In general population, the BMI during lockdown was significantly higher than that before lockdown (MD 0.94, 95% CI 0.32–1.56; *p* = 0.003). However, there was still substantial heterogeneity in the subgroup analysis (I^2^ = 84%, *p* < 0.00001).

### 3.5. Changes in BMI Classification

**Figure 4:**
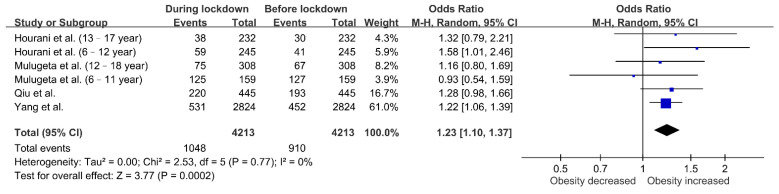
Associations between lockdown and obesity in children. The obesity rate increased during lockdown.

**Figure 5:**
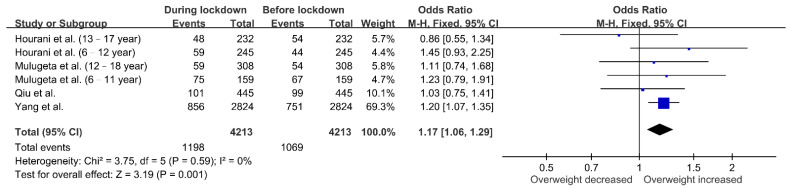
Associations between lockdown and overweight in children. The proportion of overweight children increased during lockdown.

The effect of lockdown on the proportion of overweight or obese children was assessed in four cohorts. Figure 4 shows that more children had obesity during lockdown (OR 1.23, 95% CI 1.10–1.37; *p* = 0.0002), and this result did not exhibit heterogeneity (I^2^ = 0%; *p* = 0.77). As shown in Figure 5, the forest plot also revealed an increased odds ratio of overweight in children during lockdown (OR 1.17, 95% CI 1.06–1.29; *p* = 0.001). There was no substantial heterogeneity across the studies (I^2^ = 0%; *p* = 0.59).

## 4. Discussion

To the best of our knowledge, this is the first report to quantify the effect of lockdown on the body weight of children. Our meta-analysis demonstrated an increase in body weight (MD 2.67 kg) and BMI (MD 0.77 kg/m^2^) in school-age children during the COVID-19 confinement period. The above findings explain the increased pediatric overweight and obesity revealed in subsequent analyses. China was the first country to implement lockdown as a strategy to reduce virus transmission. The prevalence of obesity increased from 10.5% to 12.6% among high school and college students during the lockdown period [23]. In one study conducted in the United States comparing the obesity prevalence between the pre-pandemic and pandemic periods, the overall obesity prevalence increased from 13.7% to 15.4% in 2020 [24]. COV-EAT, a study on children’s and adolescents’ lifestyles during lockdown in Greece, indicated that 35% of parents reported an increase in body weight among their children [2]. These studies suggest that pre-existing pediatric obesity was aggravated during the COVID-19 pandemic era.

In a recently published meta-analysis focusing on the adult population, Bakaloudi reported a 1.57-kg increase in body weight and a 0.31-kg/m^2^ increase in BMI [4]. Comparison with the current result highlights that lockdown had more impacts on pediatric body weight than adult body weight. This disparity might be partially attributed to higher daily physical activity requirements and energy needs in children. According to the 2018 Physical Activity Guidelines Advisory Committee, children and adolescents aged 6 to 17 years should perform 60 min or more of moderate-to-vigorous physical activity daily [25]. In our included studies, there were significant reductions in exercise duration [18,21,22]. In addition to decreased physical activity, children had longer periods of sedentary behavior, including playing videogames or watching television [12,13]. The out-of-school circumstances during confinement mimicked holiday or summer vacation. Studies published before the COVID-19 pandemic reported that students experience a significant increase in weight gain during summer breaks compared to during the school year [26,27].

Another factor contributing to increased body weight gain in children during the pandemic may be dietary habit changes. Significant increases were observed in the intake frequency of staple foods, sweetened beverages, sweets and desserts during lockdown in our included cohorts [12,13,28]. Multinational surveillance also revealed increased consumption of potato chips, red meat and snacks with concomitant decreases in physical activity [1,29,30]. Boredom and stress induced by COVID-19 confinement exacerbated some unhealthy eating patterns. The impact of the COVID-19 lockdown on the lifestyle behavior of children and adolescents further aggravated weight gain.

In addition to underlying health status, age contributed to the primary source of heterogeneity in our analysis. Hourani et al. reported gains of 2.3 kg in young children (6–12 years old) and 1.7 kg in older children (13–17 years old) during lockdown [13]. Mulugeta presented similar results, which revealed a higher net increase in BMI in children younger than 12 years old [17]. In one study conducted in the United States [21], the authors enrolled children with a mean age of 9.4 years, which was the youngest among our included studies, but this study reported the highest BMI increase (2.0 kg/m^2^). A similar result was also reported in one large-scale and primary care network-based study. An increase in childhood obesity was more evident in children aged 5 to 9 years than teenagers in the Philadelphia region [24]. Young children tended to be more affected during confinement according to our review. In contrast to the pattern in the general population, the disturbance associated with restrictive measures among children with pre-existing obesity tended to be lower than that among children with a normal weight according to our analysis. Nevertheless, the effect of lockdown on this population cannot be ignored. In a study exploring a weight control program, there was a significant increase in body weight during summer vacation compared to other seasons, which supports our finding [31]. Children with type 1 diabetes mellitus are another unique population. During lockdown, they had evident mood deterioration and required a higher daily total insulin dose, making glycemic control challenging [11,20]. However, the net effect on body weight or BMI could not be demonstrated in our analysis. Further study is needed. Regardless of the potential differences among subgroups, for both healthy children and children with underlying comorbidities, we need to develop individualized interventions during confinement.

The present study had some limitations. First, while we proposed a potential linkage between the pandemic and body weight gain, it is challenging to disentangle the contributions of various factors. For example, daily lifestyle, cultural background and family support might all partly contribute to the final outcome. Exploring each factor and their relationships with body weight was beyond the scope of our study. Nevertheless, our analysis across different countries showed consistent results, indicating that body weight gain during the pandemic is an emerging issue. Second, some studies in our meta-analysis were performed via online questionnaires with self-reported body weight. Inaccurate recall of previous body weight might lead to some risks of bias. However, to prevent virus transmission and maintain social distancing, bias inevitably exists in large-scale surveillance. Future studies should focus on reducing inaccuracies via study design. Third, the lockdown duration ranged from 1–5 months across the included studies. It is possible that body weight gain over time in our results was part of normal growth. Nonetheless, the mean difference in body weight gain in our analysis was 2.67 kg, which is greater than the normal 1-year growth rate of children. Additionally, the increased proportion of overweight and obese children reflected ongoing body composition changes during lockdown.

## 5. Conclusions

In our meta-analysis, we found significant increases in body weight and BMI during the lockdown period among children and adolescents. The prevalence of obesity or overweight also increased. The epidemiological burden of childhood obesity is increasing. Unfortunately, the COVID-19 pandemic worsened the epidemic. The negative effect of lockdown or restrictive measures should be carefully considered in the design or implementation of public health strategies during pandemics.

## Figures and Tables

**Table 1:** Characteristics of the included studies assessing the relationship between lockdown and body weight.

Author	Study Period	Country	Lockdown Duration in Study	Case Number	Population	Age (Mean ± SD or Range) (Year)	Male %	Method of Body Weight Measurement
Agha et al. (2021) [11]	April 2020 to June 2020	Saudi Arabia	3 months	150	Patients with type 1 diabetes mellitus	12.5 ± 4.6	28%	Direct measurement
Cipolla et al. (2021) [12]	June 2020	Italy	3 months	64	Children with obesity or overweight	13.9 ± 2.4	40.6%	Telephone interview assessing self-reported BW
Hourani et al. (2021) [13]	June 2020	Jordan	4 months	477	Healthy children	6–17 year	48.4%	Online questionnaire assessing self-reported BW
Jarnig et al. (2021) [14]	September 2019 to June 2020	Austria	4 months	764	Healthy children	9 ± 0.7	49.9%	Direct measurement
Kim et al. (2021) [15]	December 2019 to May 2020	Korea	3 months	90	Children with obesity	12.2 ± 3.4	77.8%	Direct measurement
Marigliano et al. (2021) [16]	January 2020 to June 2020	Italy	2 months	233	Patients with type 1 diabetes mellitus	13.9 ± 4.4	55.7%	Direct measurement
Mulugeta et al. (2021) [17]	March 2020 to May 2020	USA	3 months	701	Healthy children	2–18 year	44.2%	Direct measurement
Nassar et al. (2021) [18]	March 2020 to July 2020	Egypt	5 months	37	Soccer players under at home self-training	10.8 ± 0.46	100%	Direct measurement
Qiu et al. (2021) [19]	October 2019 to May 2020	China	5 months	445	Healthy children	8.7–11 year	58.4%	Direct measurement
Riso et al. (2021) [20]	January 2020 to June 2020	Italy	2 months	73	Patients with type 1 diabetes mellitus	10.8 ± 2.3	54.9%	Direct measurement
Wahl-Alexander et al. (2021) [21]	August 2019 to July 2020	USA	4 months	264	Healthy third- through eighth-grade students attending summer camp	9.6 year	49.6%	Direct measurement
Yang et al. (2020) [22]	December 2019 to February 2020	China	1 month	2824	High school students	17.5 ± 1.2	24.0%	Online questionnaire assessing self-reported BW

Abbreviations: BW, body weight; SD, standard deviation.

## Data Availability

Not applicable.

## Supplementary Materials

The following are available online at https://www.mdpi.com/article/10.3390/nu13103668/s1, Supplementary S1: The detailed search strategy for our systematic review and meta-analysis. Supplementary S2: The quality of nonrandomized studies included in the meta-analysis was assessed using the Newcastle–Ottawa Scale (NOS).
