# Supplementary material for: Weight Gain Associated with COVID-19 Lockdown in Children and Adolescents: A Systematic Review and Meta-Analysis
Source: Nutrients. 2021 Oct 19;13(10):3668. doi: 10.3390/nu13103668 (PMC8540321; doi:10.3390/nu13103668)
Supplement: Supplementary file 1 [file nutrients-13-03668-s001.zip › nutrients-1393895-supplementary/Supplementary S2-20211010.pdf]

Supplementary S2. Risk of bias assessment – Newcastle-Ottawa Scale

| | | Selection | | | | Comparability | Outcome | | | |
|---|---|---|---|---|---|---|---|---|---|---|
| Source | Study type | Representativeness of the sample | Selection of the non-exposed cohort | Ascertainment of exposure | Demonstration that outcome of interest was not present at start of study | Comparability based on design and analysis | Assessment of outcome | Was follow up long enough for outcomes to occur | Adequacy of follow up of cohorts | Total |
| Agha et al. | Cross-sectional | | * | * | * | * | * | * | * | 7 |
| Cipolla et al. | Cross-sectional | | * | * | * | * | | * | * | 6 |
| Hourani et al. | Cross-sectional | * | * | * | * | * | | * | * | 7 |
| Jarnig et al. | Cohort | * | * | * | * | * | * | * | * | 8 |
| Kim et al. | Cohort | | * | * | * | * | * | * | * | 7 |
| Marigliano et al. | Cohort | | * | * | * | * | * | * | * | 7 |
| Mulugeta et al. | Cohort | * | * | * | * | * | * | * | * | 8 |
| Nassar et al. | Cohort | | * | * | * | * | * | * | * | 7 |

| | | | | | | | | | | |
|---|---|---|---|---|---|---|---|---|---|---|
| Qiu et al. | Cohort | * | * | * | * | * | * | * | * | 8 |
| Riso et al. | Cohort | | * | * | * | * | * | * | * | 7 |
| Wahl-Alexander et al. | Cross-sectional | * | * | * | * | * | * | * | * | 8 |
| Yang et al. | Cohort | * | * | * | * | * | | * | * | 7 |
