# Supplementary material for: Weight Gain Associated with COVID-19 Lockdown in Children and Adolescents: A Systematic Review and Meta-Analysis
Source: Nutrients. 2021 Oct 19;13(10):3668. doi: 10.3390/nu13103668 (PMC8540321; doi:10.3390/nu13103668)
Supplement: Supplementary file 1 [file nutrients-13-03668-s001.zip › nutrients-1393895-supplementary/Supplementary S1-20211009.pdf]

Supplementary S1. Database Search Strategies

Search Terms genetic trait

English Synonyms	Controlled Vocabulary<br>**Emtree** / **MeSH** / **CINAHL**
coronavirus*<br>2019-nCoV*<br>"SARS Co* 2"<br>"2019 novel coronavirus*"<br>COVID*<br>"severe acute respiratory syndrome"<br>"Wuhan coronavirus*"<br>"wuhan seafood market pneumonia virus"<br>"coronavirus 2, sars"<br>"coronavirus, 2019 novel"<br>"coronavirus, wuhan"<br>"novel coronavirus, 2019"<br>"virus, covid-19"<br>"virus, sars-cov-2"<br>"disease, 2019-ncov"<br>"disease, covid-19 virus"<br>"infection, 2019-ncov"<br>"infection, covid-19 virus"<br>"infection, sars-cov-2"<br>"disease 2019, coronavirus"<br>"pandemic, covid-19"<br>"virus disease, covid-19"<br>"virus infection, covid-19"	**coronavirus disease 2019**<br>**coronaviridae infection**<br>**coronavirus infection**<br>**coronavirus**<br>**SARS-COV-2**<br>**coronavirus infections**<br>**COVID-19**<br>SARS-CoV-2<br>COVID-19<br>SARS-CoV-2<br>COVID-19 Pandemic
lockdown<br>lock*<br>"mass quarantine"<br>quarantine*<br>"cordon sanitaire"<br><br>restriction<br>measures	**Lockdown**<br>**quarantine**<br>**quarantine**<br>**stay-at-home orders**<br>**quarantine**
diet*<br><br>lifestyle*	**diet**<br>**diet**<br>diet

"life style*"<br>"factor, lifestyle"	**lifestyle**<br>**life style**<br>**life style changes**
body weight*<br>BMI<br>weight<br>body<br>"body ban mass"<br>"body mass index"<br>"quetelet index"<br>"weight, body"<br>"weights, body"<br>"body mass index"<br>"index, body mass"<br>"index, quetelet"<br>"quetelet* index"<br><br>obesit*<br>fat*<br>adiposity<br>corpulenc*<br>portliness<br>pursiness<br>adipos*<br>excess<br>overweight	**body weight**<br>**body mass**<br>**body weight**<br>**body mass index**<br>**body mass index**<br>**body weight changes**<br>**body weight**<br><br><br><br><br><br><br><br>**obesity**<br>**Obesity**<br>**Overweight**<br>**Adiposity**<br>**obesity**

Search Strategy

Database	#	Search syntax	Citations found
**Embase**	1	(Coronavirus* OR 2019-nCoV* OR "SARS Co* 2" OR "2019 novel coronavirus*" OR COVID* OR "severe acute respiratory syndrome" OR "Wuhan coronavirus*" OR "wuhan seafood market pneumonia virus" OR "coronavirus 2, sars" OR "coronavirus, 2019 novel" OR "coronavirus, wuhan" OR "novel coronavirus, 2019" OR "virus, covid-19" OR "virus, sars-cov-2" OR "disease,2019-ncov"):ti,ab,kw,de	217,091
	2	"coronavirus disease 2019"/exp OR "Coronaviridae infection"/exp OR "Coronavirus infection"/exp	172,569
	3	(Lockdown OR Lock* OR "mass quarantine" OR Quarantine* OR "cordon sanitaire" OR restrict* OR measure*):ti,ab,kw,de	5,477,500
	4	"Lockdown"/exp OR "quarantine"/exp	15,165
	5	(diet* OR lifestyle* OR "life style*" OR "factor, lifestyle" OR "health behavior" OR "physical activity"):ti,ab,kw,de	1,472,458
	6	"Diet"/exp OR "Lifestyle"/exp	502,076
	7	(body weight* OR BMI OR weight OR body OR "body ban mass" OR "body mass index" OR "Quetelet index" OR "weight, body" OR "weights, body" OR "body mass index" OR "index, body mass" OR "index, quetelet" OR "Quetelet* index" OR Obesit* OR Fat* OR adiposit* OR corpulenc* OR portliness OR pursiness OR adipos* OR excess OR overweight):ti,ab,kw,de	4,970,294
	8	"body weight"/exp OR "body mass"/exp OR "Obesity"/exp	1,475,692
	9	(#1 OR #2) AND (#3 OR #4) AND (#5 OR #6) AND (#7 OR #8) AND [embase]/lim	967
**MEDLINE**	1	(coronavirus* OR 2019-nCoV* OR "SARS Co* 2" OR "2019 novel coronavirus*" OR COVID* OR "severe acute respiratory syndrome" OR "Wuhan coronavirus*" OR "wuhan seafood market pneumonia virus" OR "coronavirus 2, sars" OR "coronavirus, 2019 novel" OR "coronavirus, wuhan" OR "novel coronavirus, 2019" OR "virus, covid-19" OR "virus, sars-cov-2" OR "disease,2019-ncov").mp	204,342
	2	exp "coronavirus"/ OR exp " SARS-COV-2"/ OR	127,679

		exp "coronavirus infections"/ OR exp "COVID-19"/	
	3	(lockdown OR lock* OR "mass quarantine" OR quarantine* OR "cordon sanitaire" OR restrict* OR measure*).mp	4,259,078
	4	exp "quarantine"/	5,245
	5	(diet* OR lifestyle* OR "life style*" OR "factor, lifestyle" OR "health behavior" OR "physical activity").mp	1,041,061
	6	exp "diet"/ OR exp "life style"/	387,479
	7	(body weight* OR BMI OR weight OR body OR "body ban mass" OR "body mass index" OR "Quetelet index" OR "weight, body" OR "weights, body" OR "body mass index" OR "index, body mass" OR "index, quetelet" OR "quetelet* index" OR obesit* OR fat* OR adiposit* OR corpulenc* OR portliness OR pursiness OR adipos* OR excess OR overweight).mp	3,392,214
	8	exp "body weight"/ OR exp "body mass index"/ OR exp "obesity"/ OR exp "overweight"/ OR exp "adiposity"/	561,869
	9	(1 OR 2) AND (3 OR 4) AND (5 OR 6) AND (7 OR 8)	606
**Cochrane CENTRAL**	1	((coronavirus* OR COVID*) OR ("2019-nCoV*" OR "SARS Co* 2" OR "2019 novel coronavirus*" OR "severe acute respiratory syndrome" OR "Wuhan coronavirus*" OR "wuhan seafood market pneumonia virus" OR "coronavirus 2, sars" OR "coronavirus, 2019 novel" OR "coronavirus, wuhan" OR "novel coronavirus, 2019" OR "virus, covid-19" OR "virus, sars-cov-2" OR "disease,2019-ncov")):ti,ab,kw	8,022
	2	[mh "coronavirus"] OR [mh "SARS-COV-2"] OR [mh "coronavirus infections"] OR [mh "COVID-19"]	1,199
	3	(lockdown OR lock* OR "mass quarantine" OR quarantine* OR "cordon sanitaire" OR restrict* OR measure*):ti,ab,kw	496,964
	4	[mh "quarantine "]	16
	5	(diet* OR lifestyle* OR "life style*" OR "factor, lifestyle" OR "health behavior" OR "physical activity"):ti,ab,kw	135,854
	6	[mh "diet"] OR [mh " life style"]	19,497

	7	((BMI OR weight OR body OR obesit* OR fat* OR adiposit* OR corpulenc* OR portliness OR pursiness OR adipos* OR excess OR overweight) OR (body weight* OR "body ban mass" OR "body mass index" OR "Quetelet index" OR "weight, body" OR 3383"weights, body" OR "body mass index" OR "index, body mass" OR "index, quetelet" OR "quetelet* index")):ti,ab,kw	300,225
	8	[mh "body weight"] OR [mh "body mass index"] OR [mh "obesity"] OR [mh "overweight"] OR [mh "adiposity"]	34,707
	9	(#1 OR #2) AND (#3 OR #4) AND (#5 OR #6) AND (#7 OR #8)	113(Trials)
	9	**Cochrane Reviews**	**3**
**CINAHL**	1	coronavirus* OR 2019-nCoV* OR "SARS Co* 2" OR "2019 novel coronavirus*" OR COVID* OR "severe acute respiratory syndrome" OR "Wuhan coronavirus*" OR "wuhan seafood market pneumonia virus" OR "coronavirus 2, sars" OR "coronavirus, 2019 novel" OR "coronavirus, wuhan" OR "novel coronavirus, 2019" OR "virus, covid-19" OR "virus, sars-cov-2" OR "disease,2019-ncov"	73,212
	2	mh ("SARS-CoV-2") OR mh ("COVID-19") OR mh ("SARS-CoV-2") OR mh ("COVID-19 Pandemic")	35.408
	3	lockdown OR lock* OR "mass quarantine" OR quarantine* OR "cordon sanitaire" OR restrict* OR measure*	957,940
	4	mh ("quarantine") OR mh ("Stay-at-Home Orders")	3,397
	5	diet* OR lifestyle* OR "life style*" OR "factor, lifestyle" OR "health behavior" OR "physical activity"	399,916
	6	mh ("diet") OR mh ("life style changes")	71,560
	7	body weight* OR BMI OR weight OR body OR "body ban mass" OR "body mass index" OR "Quetelet index" OR "weight, body" OR "weights, body" OR "body mass index" OR "index, body mass" OR "index, quetelet" OR "quetelet* index" OR obesit* OR fat* OR adiposit* OR corpulenc* OR portliness OR pursiness OR adipos* OR excess OR overweight	771,955
	8	mh ("body weight") OR mh ("body mass index") OR mh ("body weight changes")	112,697

	9	(S1 OR S2) AND (S3 OR S4) AND (S5 OR S6) AND (S7 OR S8)	244
